# Reply to Krupenko et al., Comment on “Lee et al. The Combination of Loss of ALDH1L1 Function and Phenformin Treatment Decreases Tumor Growth in KRAS-Driven Lung Cancer *Cancers* 2020, *12*, 1382”

**DOI:** 10.3390/cancers13092238

**Published:** 2021-05-07

**Authors:** Seon-Hyeong Lee, Yoon Jeon, Joon-Hee Kang, Hyonchol Jang, Kyeong-Man Hong, Dongwan Hong, Ho Lee, Soo-Youl Kim

**Affiliations:** 1 Division of Cancer Biology, Research Institute, National Cancer Center, Goyang 10408, Korea; 75461@ncc.re.kr (S.-H.L.); imyoon81@ncc.re.kr (Y.J.); 75463@ncc.re.kr (J.-H.K.); hjang@ncc.re.kr (H.J.); kmhong@ncc.re.kr (K.-M.H.); 2 Department of Medical Informatics, College of Medicine, Catholic University of Korea, Seoul 06591, Korea; dwhong@catholic.ac.kr; 3 Graduate School of Cancer Science and Policy, National Cancer Center, Goyang 10408, Korea

In the Cancers paper, we observed the increase ALDH1L1 protein expression following oncogenesis, as well as a therapeutic effect, by deleting the *Aldh1l1* gene in *Kras^LA2^* mice, a model of spontaneous non-small cell lung cancer (NSCLC) [1]. However, Prof. Sergey Krupenko and Jaspreet Sharma found some conflict within their data. We answered this disagreement with scientific data and five brief questions, including three major and two minor questions. The major questions are: Q1. Is ALDH1L1 detectable in lung cancer cells? Yes, it is detected by a specific antibody. Q2. Is ALDH1L1 protein expression high or low in cancer? It is high in some lung adenocarcinoma. We do not yet have data to conclude if this is the case in other cancers. Q3. Is ALDH1L1 expression high or low in cancer by analysis of public informatics? mRNA expression of ALDH1L1 is high in lung adenocarcinoma, but low in other cancers by analysis of TCGA data (unpublished).

**Question 1: Is ALDH1L1 detectable in lung cancer cells?**

**Yes, it is detected by a specific antibody.**

It is of utmost importance to clarify that this disagreement concerns a difference of antibody activity. We used two different antibodies to detect ALDH1L1. We used two ALDH1L1 antibodies (cat. No. ab56777 and ab175198). The catalog number of mouse monoclonal to the ALDH1L1 antibody is ab56777. The catalog number of rabbit monoclonal to the ALDH1L1 antibody is ab175198.

To detect mouse ALDH1L1 in Kras^LA2^ mice’s tissue section or tissue lysate, ab175198 must be used with the rabbit secondary antibody. To detect human ALDH1L1 in the human sample, such as human cancer cell lysate, ab56777 must be used with the mouse secondary antibody.

Otherwise, ALDH1L1 was not detected. Our results showed that Western blotting of ALDH1L1 using ab17519 had the same result as Dr. Sharma showed (Figure 1, Figure 2, Figure 3, Figure 4, Figure 5, Figure 6, Figure 7, Figure 8, Figure 9, Figure 10 and Figure 11 below, unpublished). Detailed information about western blot in Figures S1 and S2.

**Question 2: Is ALDH1L1 protein expression high or low in cancer?**

**It is high in some lung adenocarcinoma. We do not yet have data to conclude this in other cancers.**

We provided the protein expression level of ALDH1L1 by three different methods, including Western blotting (answered in Q1), MRM, and Immunohistochemical staining.

**(1) MRM:** We analyzed the expression level of ALDH1L1 by the MRM method, which is the most reliable and precise technique for quantitate protein levels. The MRM technology for ALDH analysis was introduced in the previous papers [2,3]. 

Quantification of MRM measurements. Skyline was used to quantify MRM measurements by calculating the peak areas of extracted ion chromatograms. To determine whether a peptide was detected in each cell line, we applied highly stringent criteria, including: (i) raw peak area greater than 5000, (ii) signal to noise ratio higher than 3, and (iii) coefficient of variation lower than 25% for the technical triplicates. The raw peak area values were normalized as described below. Two types of standards were used for normalization. Two peptides from human glyceraldehyde-3-phosphate dehydrogenase (Swiss-Prot accession: P04406), GALQNIIPASTGAAK and LISWYDNEFGYSNR, were used as endogenous internal standards and two peptides of E. coli β-galactosidase, IDPNAWVER, and GDFQFNISR, were used as external spiked standards. Normalized peak areas of target ALDH isoforms were calculated as follows:

$${\overset{⌣}{P}}_{i,s}=P_{i,s}\times \sqrt{\sqrt{\prod_{j=1}^{2}\frac{{\hat{n}}_{j}}{n_{j,s}}}\times \sqrt{\prod_{k=1}^{2}\frac{{\hat{x}}_{k}}{x_{k,s}}}}$$

where ${\overset{⌣}{P}}_{i,s}$ and *P_i,s_* are the normalized and raw peak areas, respectively. Of the *i*-th peptide in sample *s*, ${\hat{n}}_{j}$ is the maximum peak area of the *j*-th endogenous standard peptide over all samples in the study, *n_j,s_* is the raw peak area of the *j*-th endogenous standard peptide in sample *s*, ${\hat{x}}_{k}$ is the maximum peak area of the *k*-th exogenous spiked standard peptide over all samples, and *x_k,s_* is the raw peak area of the *k*-th exogenous spiked standard peptide in samples. All statistical data were analyzed using the R software (version 2.8.1) and Excel 2010 (version 14.0, Microsoft Office, Redmond, WA, USA) [2].

We combined two data sets from Exp Mol Med. 25 November 2016; 48 (11): e272 and Oncotarget. 2 August 2016; 7 (31): 49397–49410 [2,3], shown below.

**(2) Human lung cancer tissue array**: Immunohistochemical staining of ALDH1L1 in lung cancer tissues was statistically analyzed. 

**Analytical method:**

Tissue arrays (CC5, various human lung cancer tissues; CCN5, normal human lung tissues; *n* = 57 each case) were purchased from SuperBioChip (Seoul, Korea). Immunohistochemical staining (IHC) was performed using the UltravisionLP Detection System (Thermo Fisher Scientific Inc., Fremont, CA, USA). Briefly, after the deparaffinization of formalin-fixed, paraffin-embedded breast cancer tissues, the antigen was retrieved in 10 mM citrate buffer, pH 6.0, containing 0.05% Tween 20. After ethanol fixation, the tissues were sequentially treated with 3% hydrogen peroxide and Ultra V block solution. After 1 h of room-temperature incubation with the ALDH1L1 antibody (EMD Millipore, Princeton, NJ, USA), the slides were washed in Tris-buffered saline including Tween 20 (TBST), incubated with the primary antibody enhancer for 10 min, and exposed to the horseradish peroxidase-conjugated secondary antibody for 15 min. After re-washing in TBST, the tissue slides were incubated with diaminobenzidine chromogen (Scytek Laboratories Inc, Logan, UT) and counter-stained with Mayer’s hematoxylin (Dako Cytomation, Glostrup, Denmark). In the evaluation of ALHD1L1 expression, the staining intensity was scored on a 0-to-3 scale: 0 meaning no staining of cancer cells; 1 meaning weak staining; 2 meaning moderate staining; 3 meaning strong staining. In addition, the percentage of positive cells among cancer cells was scored. The two scores of intensity and positive stained-tumor cell percentage were multiplied, and the resulting value was used as an expression score.

**Question 3: Is ALDH1L1 expression high or low in cancer by analysis of public informatics?**

**mRNA expression of ALDH1L1 is high in lung adenocarcinoma, but low in other cancers by analysis of TCGA data.**

To investigate the gene expression level of all ALDH isotypes of human cancers, we downloaded mRNA gene expression of The Cancer Genome Atlas (TCGA) from UCSC cancer genomics browser (https:genome-cancer.ussc.edu (accessed date: 13 December 2020) [4]). Here, we showed the boxplots of a few cancers’ ALDH isotypes (a: lung adenocarcinoma, b: lung squamous cell carcinoma, c: prostate adenocarcinoma, d: stomach adenocarcinoma, e: renal clear cell carcinoma). We demonstrated that the ALDH1L1 gene expression level of cancer samples is higher than that of normal samples in non-small cell lung cancer (a. NSCLC).

**Question 4: minor comments**

(1) Minor comments demonstrated an inaccuracy regarding our statement “Bioinformatics analysis of metabolic enzymes in non-small cell lung cancer (NSCLC) revealed upregulation of aldehyde dehydrogenase (ALDH) isoforms including ALDH1L1 [5,6]”.

References showed an increase in ALDH1 activity and increase in ALDH1A1 protein level in lung cancer. In reference 8, ALDH1 refers to all subtypes of ALDH1, including ALDH1A1,1A2,1A3, 1A7, 1B1, 1L1, and 1L2, because they used the activity test instead of Western blotting. Therefore, it is better to change this to “(ALDH) isoforms including ALDH1 family [5,6]”.

(2) Comments also mentioned that there is no evidence of NADH production by ALDH1L1 in our cited reference.

**However, our statement in this discussion is correct.**

We have provided evidence which demonstrates that an increase in NADH, after over expression of ALDH1L1 by transfection, was shown in lung cancer cell lines. 

To explain our observation, we discussed NADH production in the paper. We clearly mentioned the NADPH preference of ALDH1L1 and the possible mechanism of NADH production in the discussion (below).

“*ALDH1L1* (10-formyltetrahydrofolate dehydrogenase, EC 1.5.1.6) converts 10 formyltetrahydrofolate (10-formyl-THF) to tetrahydrofolate (THF) and CO2 in an NADP+-dependent reaction [7]. The *ALDH1L1* protein is the product of a natural fusion of three unrelated genes and consequently consists of three distinct domains: formyl dehydrogenase, 10-formyl-THF hydrolase, and ALDH [7]. Like other ALDH isotypes, *ALDH1L1* performs the aldehyde dehydrogenase reaction using NADP+ or NAD+, although the Km for NAD+ is three orders of magnitude higher [8]. However, in the cytosol, the NADP+/NADPH ratio [9] is as much as three orders of magnitude lower than the NAD+/NADH ratio [10], because NADPH is abundantly supplied for anabolism, whereas NADH is rapidly oxidized to NAD+ for catabolism. Exact measurements of NADH or NADPH production by *ALDH1L1* have not been performed. NSCLC cells harboring an *ALDH1L1* knockdown produce about 10% less NADH than wild-type cells, but no change in the NADPH level was observed [3].”

**Question 5: What is the message from our Cancers paper?**

**ALDH1L1 is increased by *Kras* mutation following oncogenesis in the mice model.**

- ALDH1L1 expression was suppressed by siRNA of KRAS in NSCLC cell lines.
- ALDH1L1 expression was induced by KRAS mutation in NSCLC cell lines.
- In the spontaneous lung cancer model by Kras mutation, the ALDH1L1 protein level was increased following to the oncogenesis of adenocarcinoma.

## Figures and Tables

**Figure 1:**
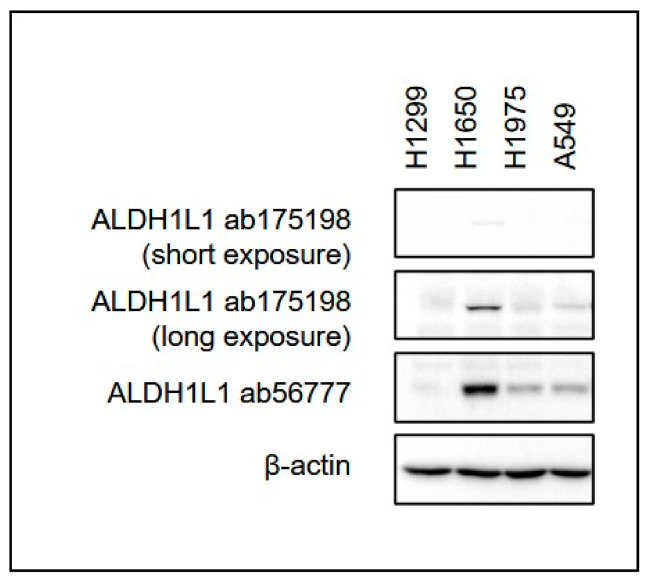
Two different antibodies against ALDH1L1 showed differential sensitivity against ALDH1L1 in various lung cancer cell lines (unpublished).

**Figure 2:**
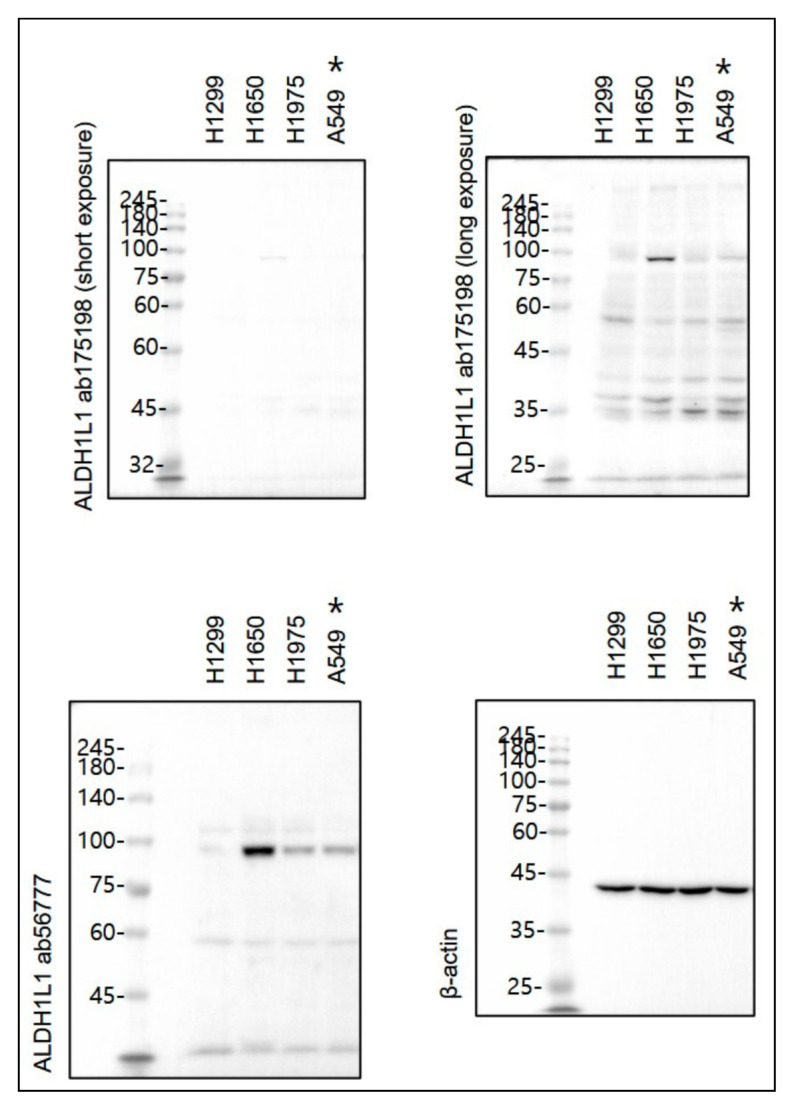
We tested 2 antibodies of ab56777 and ab175198. ALDH1L1 protein was not detected in A549 cells using ALDH1L1 ab175198 antibody, but ALDH1L1 protein was detected in A549 cells using ALDH1L1 ab56777 antibody (unpublished).

**Figure 3:**
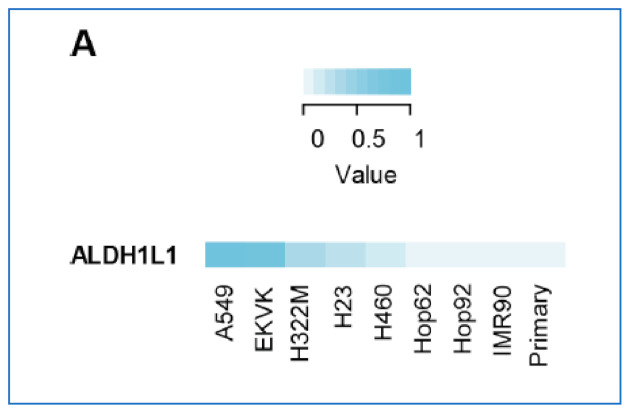
Expression of ALDH1L1 in lung cancer cell lines was measured by multiple reaction monitoring mass spectrometry (MRM-MS) [3].

**Figure 4:**
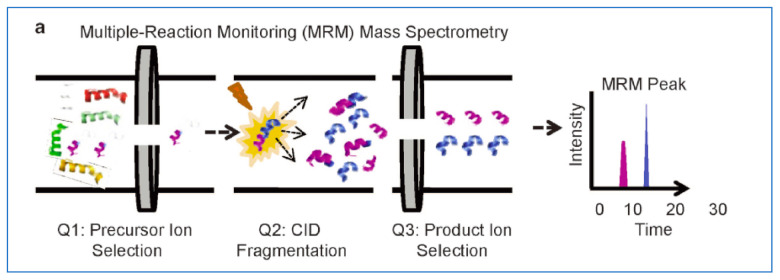
Schematic diagram of liquid chromatography multiple reaction-monitoring mass spectrometry (LC-MRM-MS) [2].

**Figure 5:**
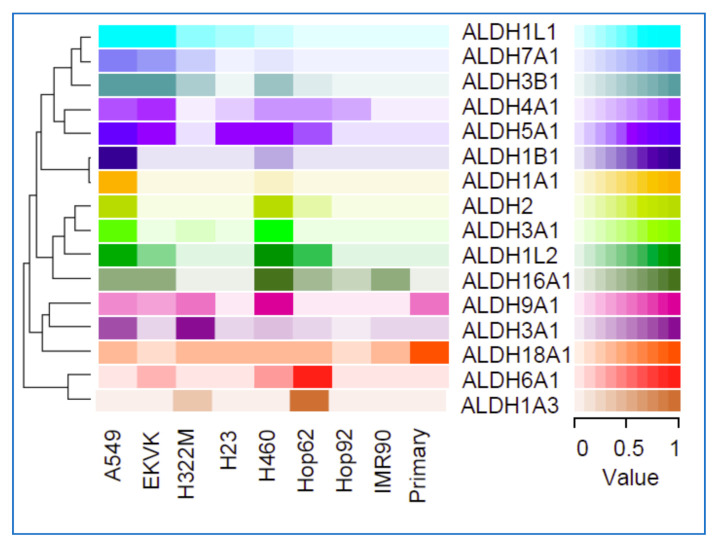
Expression of ALDH isoforms in lung cancer cell lines was measured by multiple reaction monitoring mass spectrometry (MRM-MS) [3].

**Figure 6:**
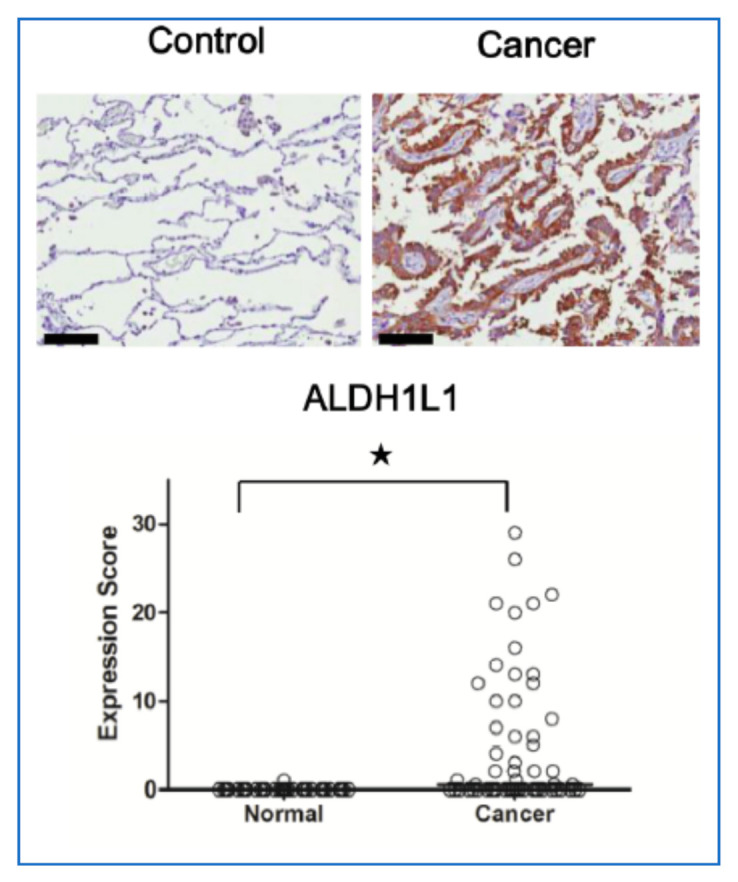
Representative immunohistochemical staining of ALDH1L1 in normal and cancerous lung tissue. Scale bar = 100 μm. Expression of ALDH1L1in cancerous (cancer) and normal lung type I and II pneumocytes (control). ★ *p* < 0.001, *n* = 57 for each case [3].

**Figure 7:**
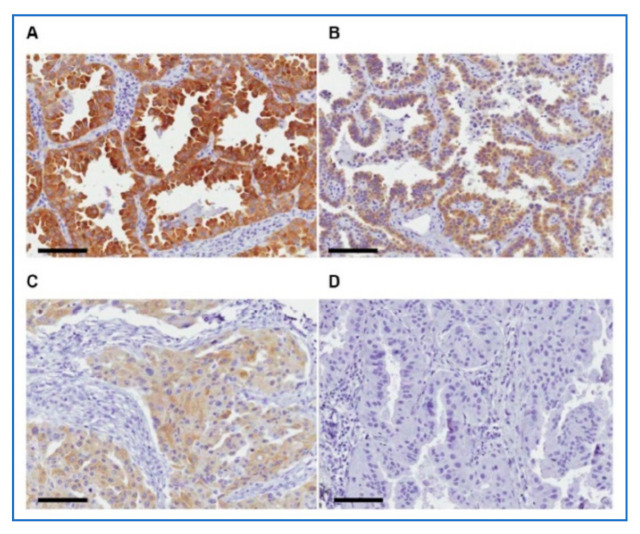
Supplementary Figure S1: Expression pattern of ALDH1L1 in lung cancer. (**A**) strong expression (**B**) moderate expression. (**C**) weak expression. (**D**) negative expression. Scale bar = 100 μm.

**Figure 8:**
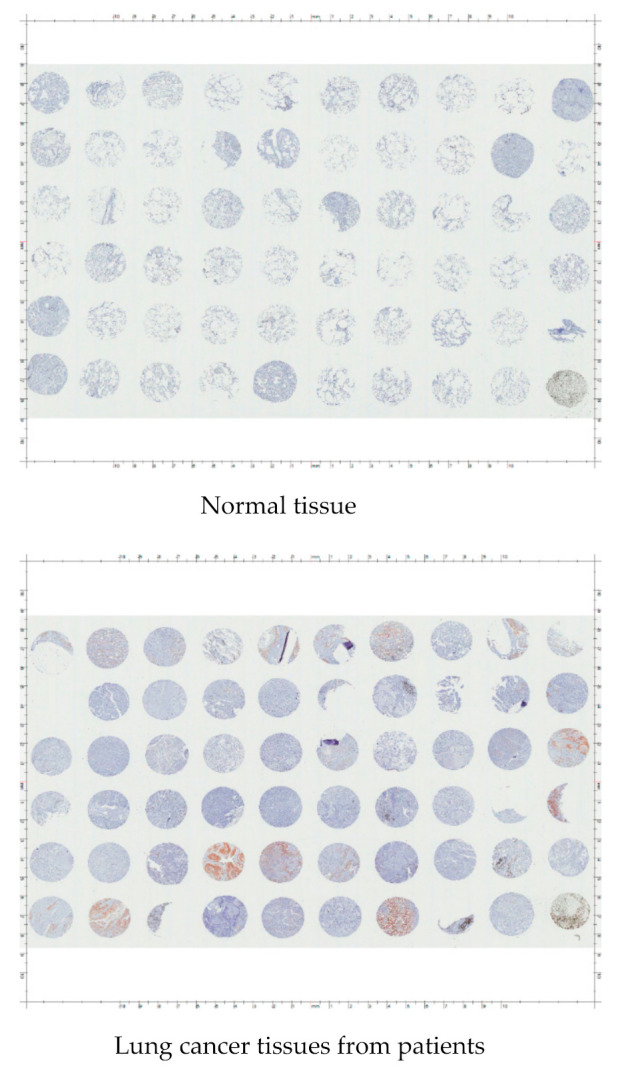
Raw data from immunohistochemical staining of ALDH1L1 using tissue array.

**Figure 9:**
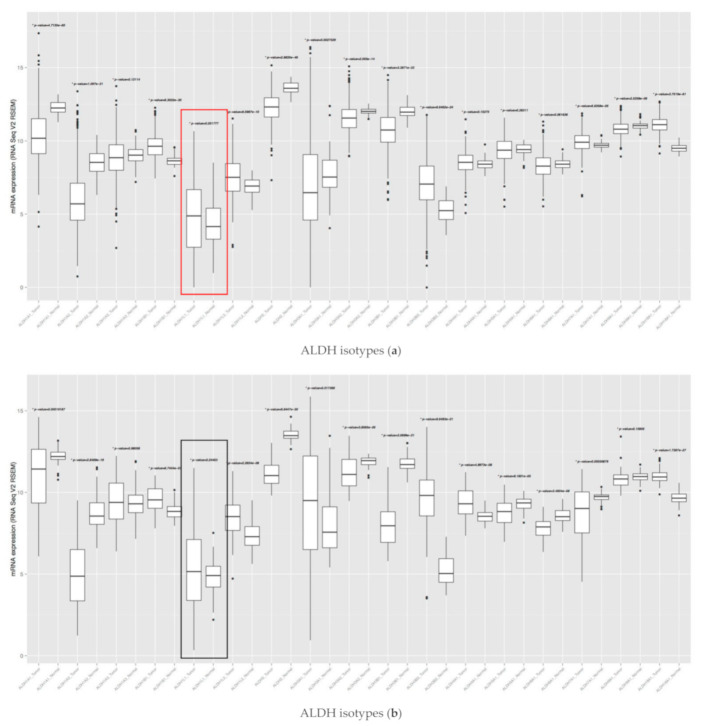

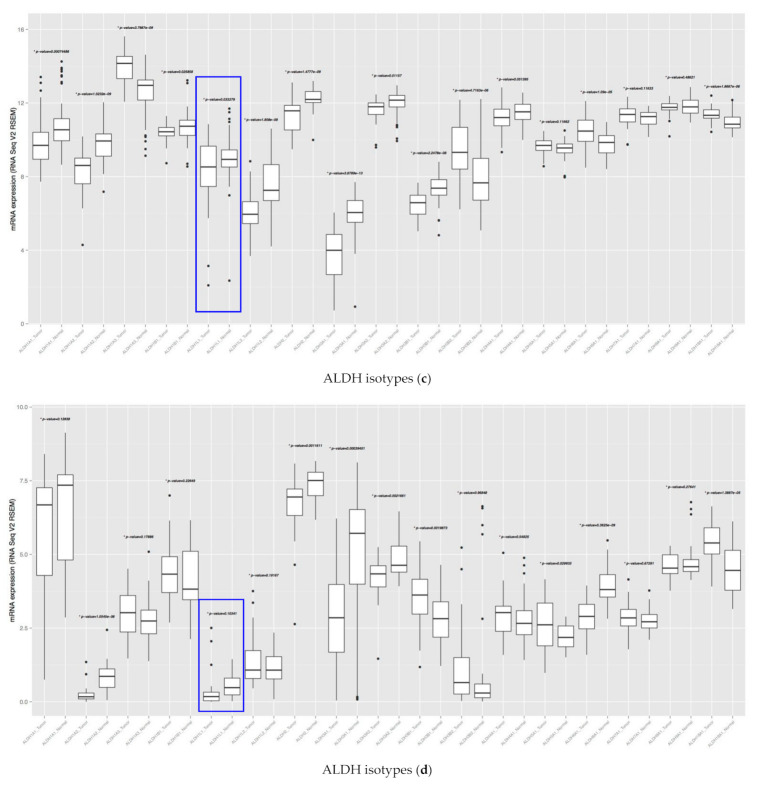

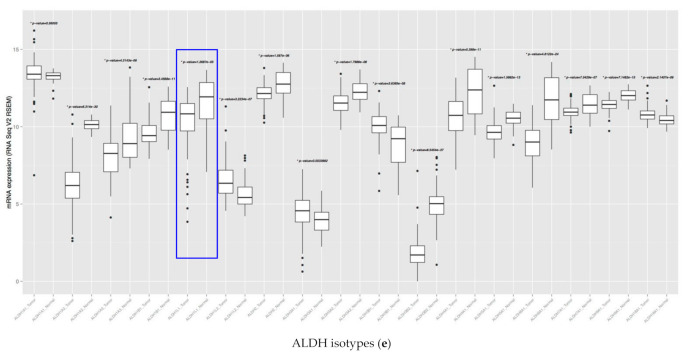
mRNA levels of ALDH isozymes. (**a**) mRNA expression of ALDH1L1 (red box, high in tumor): lung adenocarcinoma. (**b**) mRNA expression of ALDH1L1 (black box, no change in tumor): lung squamous cell carcinoma. (**c**) mRNA expression of ALDH1L1 (blue box, low in tumor): prostate adenocarcinoma. (**d**) mRNA expression of ALDH1L1 (blue box, low in tumor): stomach adenocarcinoma. (**e**) mRNA expression of ALDH1L1 (blue box, low in tumor): renal clear cell carcinoma.

**Figure 10:**
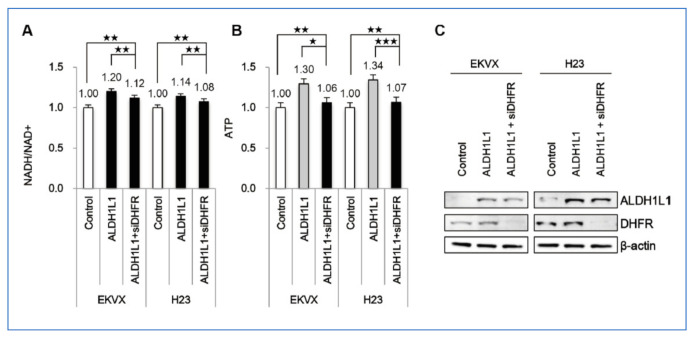
Effect of ALDH1L1 over expression on induction of NADH and ATP production. (**A**–**C**) EKVX and H23 cells were transfected with plasmid expressing ALDH1L1 for 24 h and incubated with siRNA of DHFR for 24 h [3]. ★ *p* < 0.05, ★★ *p* < 0.01, ★★★ *p* < 0.001

**Figure 11:**
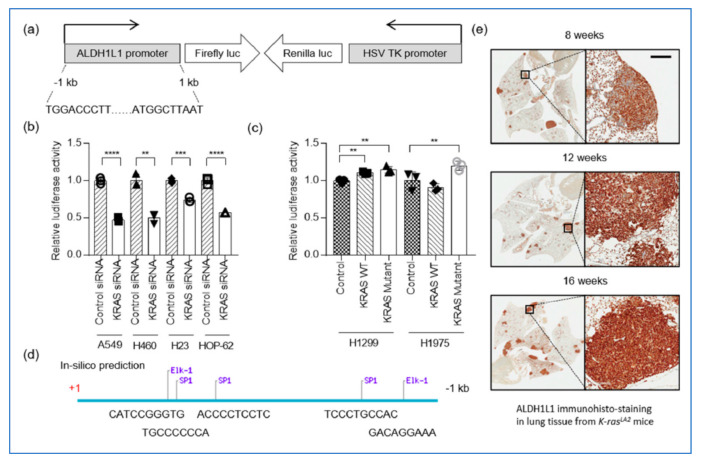
ALDH1L1 is a target of oncogenic KRAS. (**a**) Schematic representation of the reporter construct used in the cell-based transduction system. This lentiviral reporter construct expressed firefly luciferase under the control of the ALDH1L1 promoter and Renilla luciferase under the control of the HSV TK promoter. (**b**) KRAS-mutant NSCLC cells stably expressing the reporter system described in (**a**) were treated with control or KRAS siRNA. Firefly and Renilla luciferase activities were normalized against the corresponding levels in the sample transfected with control siRNA. Bars show relative luciferase activity (*n* = 3). (**c**) KRAS WT NSCLC cells stably expressing the reporter system described in (**a**) were transfected with control (empty vector), KRAS WT, or KRAS mutant (G12D). Firefly and Renilla luciferase activities were normalized against the corresponding levels in the sample transfected with empty vector. Bars indicate relative luciferase activity (*n* = 3). (**d**) In silico prediction of binding sites for transcription factors downstream of KRAS: SP1, transcription factor Sp1; Elk-1, ETS Like-1 transcription factor. (**e**) Immunohistochemical staining of ALDH1L1 in lungs isolated from Kras^LA2^ mice at 8, 12, and 16 weeks of age. The scale bar represents 200 mm (** *p* < 0.01, *** *p* < 0.001 and **** *p* < 0.0001) [1].

## Supplementary Materials

The following are available online at https://www.mdpi.com/article/10.3390/cancers13092238/s1, Figure S1: Detailed information about western blot in Figure 1, Figure S2: Detailed information about western blot in Figure 3.
