# Supplementary material for: Reply to Krupenko et al., Comment on “Lee et al. The Combination of Loss of ALDH1L1 Function and Phenformin Treatment Decreases Tumor Growth in KRAS-Driven Lung Cancer Cancers 2020, 12, 1382”
Source: Cancers (Basel). 2021 May 7;13(9):2238. doi: 10.3390/cancers13092238 (PMC8124425; doi:10.3390/cancers13092238)
Supplement: Supplementary file 1 [file cancers-13-02238-s001.zip › cancers-1061700-supplementary.pdf]

Supplementary Materials

The combination of loss of ALDH1L1 function and phenformin treatment decreases tumor growth in *KRAS*-driven lung cancer

Seon-Hyeong Lee, Yoon Jeon, Joon Hee Kang, Hyonchol Jang, Kyeong Man Hong, Dongwan Hong, Ho Lee and Soo-Youl Kim

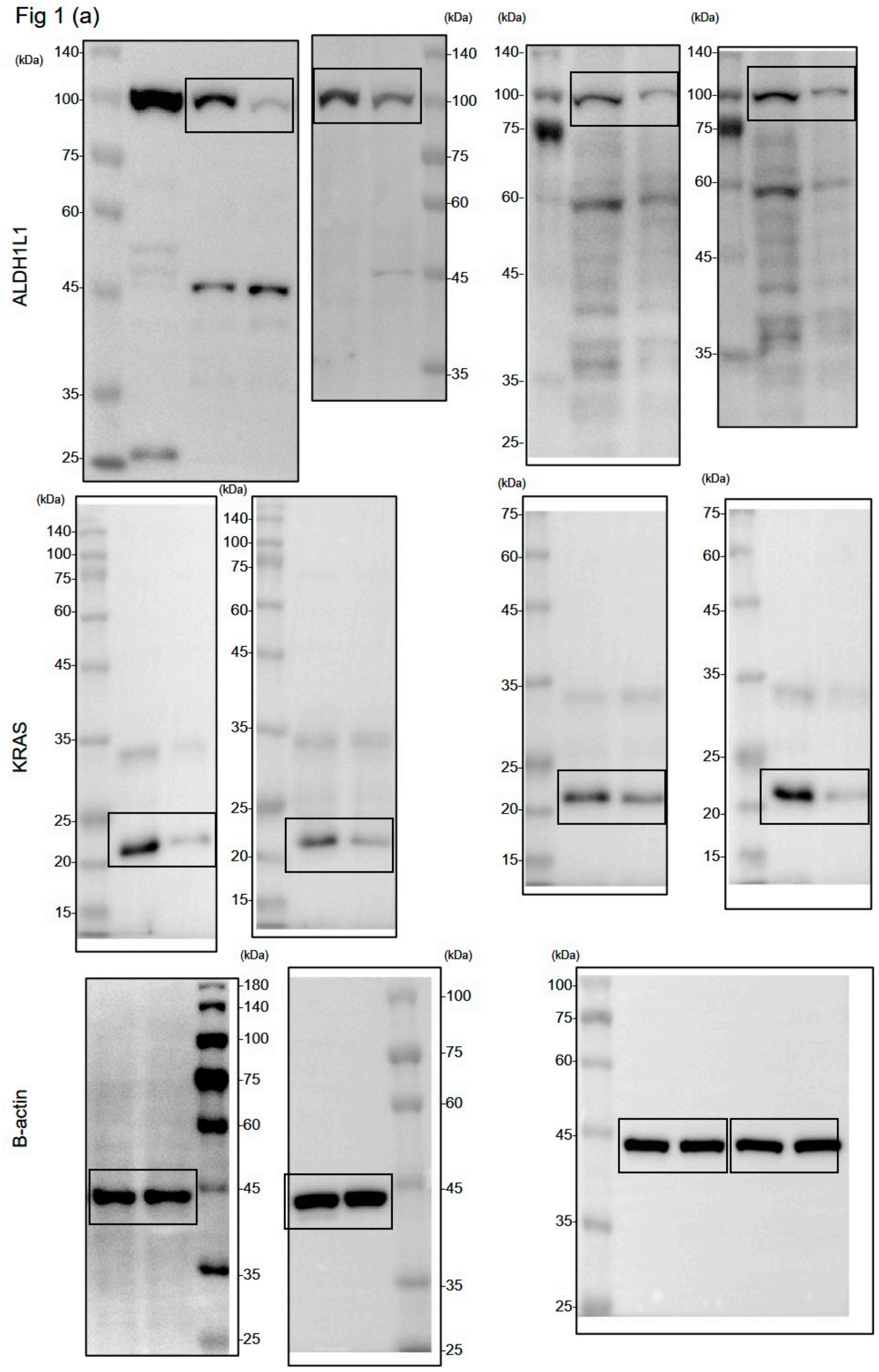

Figure S1. Detailed information about western blot in Figure 1.

Fig 3 (b)

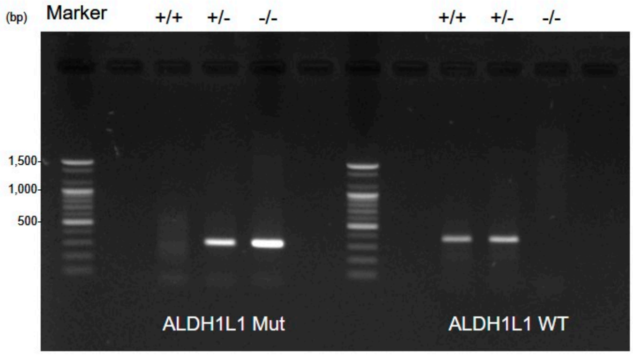

Fig 3 (c)

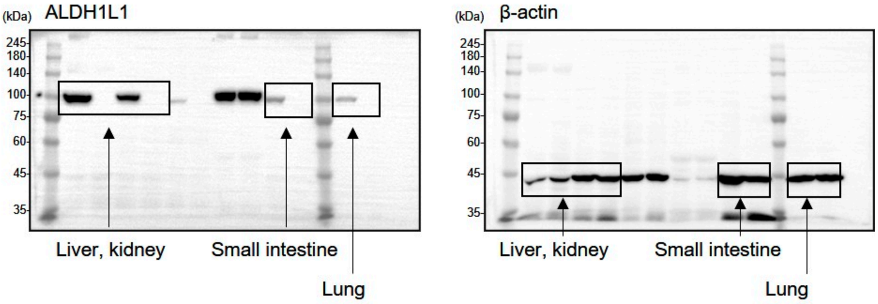

Figure S2. Detailed information about western blot in Figure3.
